# No Differences in Rotational Thromboelastometry Measurements between Portal and Peripheral Circulation in Cirrhotic Patients Undergoing TIPS

**DOI:** 10.3390/jpm13030424

**Published:** 2023-02-26

**Authors:** Sotiria Bedreli, Paul Manka, Matthias Buechter, Michael Jahn, Jens M. Theysohn, Ali Canbay, Antonios Katsounas

**Affiliations:** 1 Department of Internal Medicine and Gastroenterology, Marienhospital Gelsenkirchen, 45886 Gelsenkirchen, Germany; 2 Department of Medicine, University Hospital Knappschaftskrankenhaus Bochum, 44892 Bochum, Germany; 3 Faculty of Medicine, Ruhr University Bochum, 44901 Bochum, Germany; 4 Department of Medicine and Gastroenterology, Katholische Kliniken im Märkischen Kreis gem, GmbH, 58638 Iserlohn, Germany; 5 Department of Nephrology, Alfried-Krupp Krankenhaus Essen, 45131 Essen, Germany; 6 Department of Diagnostic and Interventional Radiology and Neuroradiology, University Hospital Essen, University Duisburg-Essen, 45122 Essen, Germany; 7 Division Infectious Diseases and Critical Care Medicine, Department of Medicine, University Hospital Knappschaftskrankenhaus, 44892 Bochum, Germany

**Keywords:** ROTEM^®^, TIPS, liver disease, thrombosis risk assessment, hypercoagulable state, portal hypertension, hepatic decompensation

## Abstract

Background: In patients with liver cirrhosis, transjugular intrahepatic portosystemic shunt (TIPS) is considered a standardized treatment of refractory ascites or variceal bleeding. TIPS thrombosis (TT) and/or portal vein thrombosis (PVT) are possible complications during/after TIPS placement. Previous studies suggested increased clotting activity in portal circulation (PORC). This pilot study aimed to evaluate alterations and differences of coagulation function in PORC and in peripheral circulation (PERC) via rotational thromboelastometry during TIPS. Methods: Blood samples were collected from cirrhotic patients (*n* = 13; median Model of End Stage Liver Disease, MELD Score: 12; median age: 60 years) undergoing TIPS (10/13 TIPSs were elective procedures due to refractory ascites) as follows: median cubital vein (MCV; PERC)—confluence of the three hepatic veins to the inferior cava vein (HV/ICV; PORC)—portal vein (PV; PORC)—TIPS (PORC). This research utilized four variables of the extrinsic test EXTEM, i.e., clotting time (CT), clot formation time (CFT), maximum clot firmness (MCF), and maximum lysis (ML). Results: EXTEM results [mean, M (range) ± standard deviation, SD (range)] showed no significant differences for CT [M (70–73) ± SD (9–13); *p* = 0.93] or CFT [M (137–155) ± SD (75–112); *p* = 0.97] or MCF [M (51–54) ± SD (9–10); *p* = 0.90] or ML [M (9–10) ± SD (4–5); *p* = 0.89] between the compartments, i.e., MCV vs. HV/ICV vs. PV vs. TIPS. Overall, we detected no differences in coagulation function between PERC and PORC. Conclusion: These results are in contrast to previous reports suggesting increased clotting activity in PORC vs. PERC in association with liver cirrhosis. Rotational thromboelastometry-based evaluation of coagulation function in PERC appears to reliably reflect coagulation function in PORC with respect to risk estimation for TT and/or PVT in cirrhotic patients undergoing TIPS.

## 1. Introduction

In patients with end-stage liver disease, transjugular intrahepatic portosystemic shunt (TIPS) implantation is considered a standardized intervention for treatment of complications due to portal hypertension, such as refractory ascites [1,2], variceal bleeding [1,3], or other severe conditions, e.g., Budd-Chiari syndrome [1,4,5], hepatorenal syndrome, and hepatic hydrothorax [1,6]. However, TIPS remains one of the most challenging angiographic techniques, requiring high expertise to limit procedure- and/or shunt-associated complications [7]. Thrombosis is a post-TIPS complication that occurs in up to 10% of cases [8]. TIPS thrombosis (TT), which usually emerges within days after TIPS intervention or occasionally also during deployment, is often attributable to graft misplacement and/or underlying hypercoagulable pathologies [9,10], which may remain undetectable in routine screening tests [11]. Although molecular pathomechanisms accounting for the recently recognized hypercoagulable state in liver cirrhosis—favoring TT and/or portal vein thrombosis (PVT)—have not been completely elucidated yet [12], there is evidence that bacterial endotoxins predispose to thrombotic complications in the portal circulation (PORC) via tissue factor up-regulation, thereby increasing generation of thrombin [13]. In fact, previous research reported significantly higher endotoxin concentrations in the PORC in comparison to the peripheral circulation (PERC) [14,15]. Moreover, earlier studies investigating the potential interplay between clotting activation and endotoxins in cirrhotic patients undergoing TIPS suggested that thrombin generation and D-dimers were increased in the PERC of cirrhotic patients compared to controls [15,16]. According to the same sources, the grade of thrombin generation and hyperfibrinolysis, which correlated with the grade of endotoxemia, was higher in the PORC versus the PERC [14,15]. These findings suggest that specific conditions ruling the gut-liver-axis, such as increased endotoxemia in the PORC, which aggravates with worsening liver disease, among other possibly yet unidentified factors, may represent key mechanisms of overstimulation of clotting processes that favor TT and/or PVT [17]. However, to the best of our knowledge, coagulation pathway activation in the PORC—versus PERC—has never been evaluated using rotational thromboelastometry or other viscoelastic tests in patients with liver cirrhosis.

During the last two decades, viscoelastic tests using whole blood specimens have revolutionized our understanding in hemostaseology as, in contrast to conventional tests, they can evaluate coagulation dynamics from clot formation to clot lysis [11,18]. Especially in the field of clinical hepatology, rotational thromboelastometry has achieved acceptance as a feasible and reliable point-of-care tool, not only for differential hemostatic management during hemorrhages, but also for bleeding or thrombosis risk assessment [19,20].

Against this background, this analysis aimed to investigate and characterize coagulation profiles in PORC—in comparison to PERC—in patients with liver cirrhosis undergoing TIPS implantation using rotational thromboelastometry and check for potential associations with occurring TT and/or PVT within 30 days post-intervention.

## 2. Materials and Methods

### 2.1. Patients

Patients with liver cirrhosis (*n* = 13) treated in the Intermediate Care Unit of the Department of Gastroenterology and Hepatology at the University Hospital Essen were subjected to this analysis within twelve months (2016–2017). Patients’ demographics and laboratory values are summarized in Table 1. 

### 2.2. Study Design

This project, which was conceived as a monocentric pilot cohort study, included patients with liver cirrhosis (*n* = 13) undergoing TIPS implantation. Patients were assigned into three categories (cirrhosis: mild vs. moderate vs. severe) according to the Child-Pugh (CP) Score or into two categories (cirrhosis: mild/moderate vs. severe) according to the Model of End Stage Liver Disease (MELD) Score [21]:

- mild/moderate liver cirrhosis: MELD < 15 or CP 5–6 points [stage A: mild cirrhosis] or CP 7–9 points [stage B: moderate cirrhosis]
- severe liver cirrhosis: MELD ≥ 15 or CP 10–15 points [stage C]

The majority of patients with MELD < 15 (i.e., 8 of 9 patients) had a moderate cirrhosis (CP stage B). Furthermore, a median MELD score of 12 and a median CP score of 8 (i.e., stage B) has been calculated for thirteen patients included in this pilot study. 

According to research protocol, patients were followed up for 30 days from the day of TIPS implantation regarding potential development of TT and/or PVT. Endpoint was to investigate and characterize coagulation profiles in the PERC in comparison to the PORC in patients with liver cirrhosis using rotational thromboelastometry (ROTEM^®^, ©Tem Innovations GmbH, Martin-Kollar-Strasse 13–15, D-81829, Munich, Germany) and check for potential associations with TT and/or PVT. 

### 2.3. Blood Sampling

Patients (*n* = 13), whose blood samples were included in final analysis, were selected as a “convenience subset” by means of on-site availability (i.e., available vs. not available) of the respective ROTEM^®^ delta device during TIPS implantation. For additional information and further clarification, readers are directed to the Discussion section.

PERC blood samples were collected from the median cubital vein (MCV). PORC blood samples were withdrawn from the prehepatic venous compartment, i.e., the portal vein (PV), from inside the stent lumen during the TIPS placement (TIPS), and from the posthepatic venous system at the level of the confluence of three hepatic veins to the inferior cava vein (HV/ICV). Blood sampling occurred in the following order: MCV, HV/ICV, PV, TIPS. 

Overall, blood sampling was performed using a 5.4 mL Coagulation SARSTEDT Monovette^®^ (Sarstedt, Germany). All PORC samples were collected as rest materials following standard TIPS protocol that foresees X-ray control and aspiration of the catheter as two safeguards used for placing the catheter in an optimal position; the use of this TIPS protocol was approved by the local institutional review board. PERC blood samples were obtained from an 18-G cannula that was routinely placed in the left MCV to ensure safe circulation management. Finally, all samples were subjected to ROTEM^®^ analysis.

### 2.4. Rotational Thromboelastometry

According to the manufacturer’s instructions, we chose the ROTEM^®^ delta device to perform the coagulation assay EXTEM [22]. In this extrinsic screening test, coagulation was triggered by 20 µL tissue factor and 20 µL CaCl2 0.2 mol/l. ROTEM^®^ values included clotting time (CT), clot formation time (CFT), maximum clot firmness (MCF), and maximum lysis (ML). Reference values have been defined according to the reference range stated by the manufacturer (©Tem Innovations GmbH, Martin-Kollar-Strasse 13–15, D-81829, Munich, Germany). Evaluation of ML was performed after 60 min.

### 2.5. Statistical Analysis

**Figure 1:**
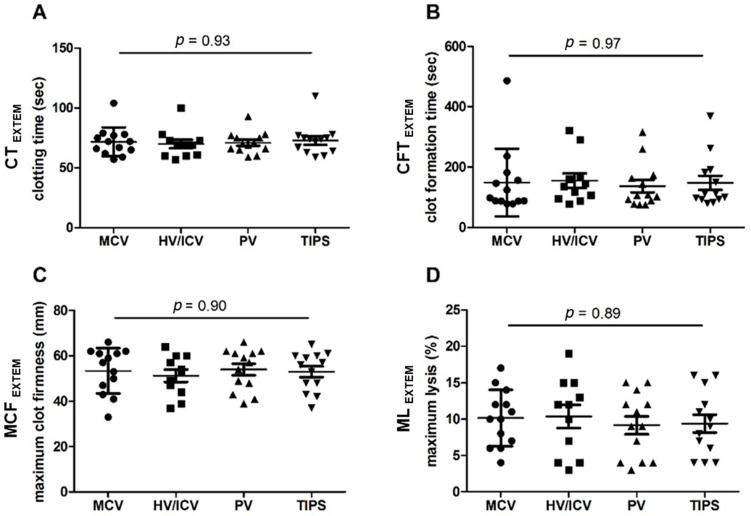
EXTEM/CT (**A**), EXTEM/CFT (**B**), EXTEM/MCF (**C**), and EXTEM/ML (**D**) measurements during TIPS implantation in patients with liver cirrhosis (*n* = 13) in peripheral (MCV) vs. pre-hepatic (PV, TIPS) vs. post-hepatic (HV/ICV) blood samples. Results are presented as mean ± standard deviation. Clotting time (CT); clot formation time (CFT); maximum clot firmness (MCF); maximum lysis (ML): reduction of the clot firmness (%) after MCF (in relation to MCF). Seconds (s); millimeters (mm). Median cubital vein (MCV); confluence of three hepatic veins to the inferior cava vein (HV/ICV); portal vein (PV); transjugular intrahepatic portosystemic shunt (TIPS).

ROTEM^®^ measurements are presented as median (minimum to maximum, min; max; Table 2) or mean (±standard deviation, SD; Figure 1) values. Comparison of ROTEM^®^ values (mean ± SD) between the sample groups, i.e., MCV vs. HV/ICV vs. PV vs. TIPS, was performed using the one-way analysis of variance (ANOVA). Overall, *p* < 0.05 was considered significant. Calculations and graphs were generated using GraphPad version 5.00 for Windows, GraphPad Software, San Diego, CA, USA.

## 3. Results

### 3.1. Demographic Data

This pilot study included thirteen patients with liver cirrhosis, who underwent TIPS (Table 1). The most common indication for TIPS was refractory ascites (*n* = 10, 76.9%). The median age was 60 (min.: 22; max.: 74) years. Liver disease severity assessment revealed a median MELD Score of 12 (min.: 8; max.: 22). Median INR was 1.12 (min.: 1.05; max.: 1.41) and median fibrinogen concentration was 269 mg/dL (min.: 101; max.: 627). Most of the patients had thrombocytopenia due to portal hypertension and splenomegaly with a median platelet count/nL of 112 (min. 29; max.: 259). 

During the follow-up time of 30 days after TIPS, none of the patients developed TT and/or PVT.

### 3.2. TIPS Implantation and Procedure Related Data

Covered stents were placed in 13 (100%) patients. The stent diameters were 10 mm (*n* = 1, 7.8%), 8 mm (*n* = 7, 53.8%), and 6 mm (*n* = 5, 38.4%). The mean stent length was 7.8 ± 0.4 cm. The following lengths were used: 7 and 8 cm in 3 and 10 patients, respectively. Each stent was 2 cm longer in full length; this portion was uncovered. An adjunctive stent of 5 cm (+2 cm) was required to cover long track in one patient who initially received a stent of 7 cm (+2 cm).

The portal pressure gradient (PPG) was reduced from a mean of 22.1 ± 6.7 mm Hg to 8.9 ± 2.4 mm Hg. The mean decrease in post-TIPS PPG was 13.2 ± 4.2 mm Hg. Recurrent bleeding after TIPS placement did not occur during follow-up in any of the patients undergoing emergency TIPS due to variceal bleeding. One month after TIPS placement, complete or partial ascites response was seen in 100% of the cirrhotic patients (*n* = 10) undergoing TIPS due to refractory ascites. Gut decontamination was performed with oral lactulose (10–30 mL three times per day) after TIPS implantation for at least 7 days in all patients. Patients undergoing emergency TIPS due to variceal bleeding (*n* = 3) also received Ceftriaxon (2 g/24 h) intravenously for 7 days.

### 3.3. EXTEM Analysis in Blood Samples from Different Vascular Compartments during TIPS 

EXTEM analysis showed no statistically significant differences for clotting time (CT, *p* = 0.93; Figure 1A), clot formation time (CFT, *p* = 0.97; Figure 1B), maximum clot firmness (MCF, *p* = 0.90; Figure 1C), or maximum lysis (ML, *p* = 0.89; Figure 1D) between the compartments, i.e., MCV vs. HV/ICV vs. PV vs. TIPS. Figure 1A–D shows mean ± standard deviation of CT, CFT, MCF, and ML:

- Mean CT was 72 ± 12 s in MCV blood samples, 70 ± 12 s in HV/ICV blood samples, 71 ± 9 s in PV blood samples, and 73 ± 13 s in TIPS blood samples.
- Mean CFT was 149 ± 112 s in MCV blood samples, 155 ± 80 s in HV/ICV blood samples, 137 ± 75 s in PV blood samples, and 148 ± 85 s in TIPS blood samples.
- Mean MCF was 53 ± 10 mm in MCV blood samples, 51 ± 9 mm in HV/ICV blood samples, 54 ± 9 mm in PV blood samples, and 53 ± 9 mm in TIPS blood samples.
- Mean ML was 10 ± 4% in MCV blood samples, 10 ± 5% in HV/ICV blood samples, 9 ± 4% in PV blood samples, and 9 ± 4% in TIPS blood samples.

In addition, medians were not significantly different between the compartments, i.e., MCV vs. HV/ICV vs. PV vs. TIPS. Median (min; max) as well as normal range values for CT, CFT, MCF, and ML are summarized in Table 2.

## 4. Discussion

This analysis, which has been based on four thromboelastometric variables in EXTEM, i.e., CT, CFT, MCF, and ML, detected no differences in coagulation function between blood samples withdrawn from the PERC versus the PORC in a convenience subset of patients with liver cirrhosis. These findings are in contrast to previous reports which suggest overall increased clotting activity in the PORC relative to the PERC in cirrhotic patients [14,15]. Thus, using rotational thromboelastometry, evaluation of coagulation function in peripheral blood—before TIPS implantation—appears to reliably depict coagulation function in portal blood among patients with liver cirrhosis. 

As none of the thirteen TIPS recipients developed TT or PVT within 30 days post-intervention, this research failed to investigate the relation between potentially exaggerated coagulation in the PORC and thrombotic complications during and/or after TIPS in the setting of liver cirrhosis. In fact, based on consistent EXTEM measurements via CT and MCF in PERC and PORC blood samples, none of these thirteen patients was evaluated as being at risk for TT and/or PVT. Clearly, thirteen—by means of convenience sampling selected—patients with liver disease who have shown no prothrombotic alterations (and no significant differences) according to EXTEM within (or between) the PORC and the PERC, have indeed not developed TIPS thrombosis. Considering that none of these thirteen patients had received anticoagulant therapy before TIPS, these limited (but consistent) results may actually support causality. Again, based on rotational thromboelastometry, our data do not support the scenario of different coagulation states between the PERC and the PORC in patients with liver cirrhosis.

For the purposes of this study, we only applied EXTEM because this test delivers the most informative results of ROTEM^®^. EXTEM evaluates function of the extrinsic pathway, whereas EXTEM/CT provides information similar to the prothrombin time. EXTEM/MCF, which depends on platelets and/or fibrinogen concentration and/or function, evaluates clot stability. EXTEM/CFT, which also depends on platelets and fibrinogen, evaluates clot propagation. EXTEM/ML corresponds to the stability of the blood clot against fibrinolytic processes. In line with this approach, previous research also assessed coagulation activation, clot formation, and fibrinolysis by applying EXTEM as the main screening test [23]. 

As expected, application of oral lactulose for gut decontamination towards reduction of the risk of infection and/or hepatic encephalopathy after TIPS was effective in our cohort [24]. Intravenous antibiotic therapy with Ceftriaxon for 7 days was administered according to the guidelines for gastrointestinal bleeding in cirrhosis that can be found in the Baveno Statement [25]. Against this background no complications in terms of infection, recurrent bleeding and/or hepatic encephalopathy occurred among the study participants within 4 weeks after TIPS. 

To the best of our knowledge, the present monocentric pilot study has been the first to evaluate coagulation activity in the PORC—versus PERC—using rotational thromboelastometry in patients with liver cirrhosis. However, it has various limitations that warrant special attention. First, these findings may be of limited statistical validity due to the small sample size of our cohort. Second, rotational thromboelastometry neglects the role of the vascular endothelium during the coagulation process [26]. Third, due to limited on-site availability of ROTEM^®^ delta devices and the mandatory prioritization of critical emergencies, e.g., hemorrhages over measurements for research purposes, we have been able to timely perform rotational thromboelastometry tests (with blood samples collected as safeguards from the PORC and/or rest materials from the PERC) only in approximately 40% of all patients that underwent TIPS within 12 months (2016–2017). Most prominently, limitations of this study include that no patients developed TT or PVT, and thus, its validity in external populations of patients with liver cirrhosis undergoing TIPS remains to be further investigated. Moreover, all ROTEM measurements were performed by convenience sampling. Consequently, patients (*n* = 13) included in the final analysis should be considered a “convenience subset”. Furthermore, as a single center research, a higher risk of bias may apply versus other multicenter trials. These issues should be addressed in larger cohorts with unbiased recruitment in the future. 

Taken together, the herein presented methodic approach, once validated in larger studies, may provide a useful tool for individualized risk estimation for TT and/or PVT in patients with liver cirrhosis undergoing TIPS.

## 5. Conclusions

Further efforts to gain more differentiated insights into coagulation physiology in the PORC in comparison to the PERC—by means of viscoelastic tests such as ROTEM^®^—may lead to the establishment of personalized and perhaps more accurate thrombosis risk assessment and treatment strategies. Thus, we hope to stimulate the interest of the scientific community for more intense research on regulation of coagulation pathways within the gut-liver axis.

### Ethical Approval

This research was performed in accordance with the Declaration of Helsinki of 1975, as revised in 2008, and the guidelines of the International Conference for Harmonization for Good Clinical Practice and was approved by the local institutional review board (IRB: Ethik Kommission am Universitätsklinikum Essen; IRB protocol no. 18-8184-BO). The IRB has found and documented that this research involves no more than minimal risk to the subjects; therefore, the IRB waived the requirements to obtain additional informed consents for collection of PORC blood samples since this step has been executed as a part of standard medical aid practices and/or as rest materials.

## Figures and Tables

**Table 1:** Demographic characteristics and clinical parameters of patients undergoing TIPS. Data are presented as median (min; max) or as absolute count “*n*” with percentage (%).

Characteristics	TIPS; *n* = 13	Normal Range
Underlying liver disease (*n*) Primary sclerosing cholangitis (PSC) Alcoholic steatohepatitis (ASH) Hepatitis C virus (HCV) Autoimmune hepatitis Non-alcoholic steatohepatitis (ASH) Cryptogenic cirrhosis	1 (8%) 6 (46%) 1 (8%) 2 (15%) 2 (15%) 1 (8%)	-
Sex (*n*) male female	11 (85%) 2 (15%)	-
Age (years)	60 (22; 74)	-
MELD ^§^ Score ≥15 points <15 points	12 (8; 22) 4 (31%) 9 (69%)	-
Child-Pugh Score [Classification] 5–6 points [stage A] 7–9 points [stage B] 10–15 points [stage C]	8 (6; 11) 1 (8%) 8 (61%) 4 (31%)	-
aPTT ^‡^ (s)	29 (26; 66)	24.4–32.4
Thrombocytes (cells/nL)	112 (29; 259)	140–320
INR ^†^	1.12 (1.05; 1.41)	-
Fibrinogen (mg/dL)	269 (101; 627)	180–350
Hemoglobin (g/dL)	10.6 (6.6; 14.5)	13.7–17.2
Bilirubin total (mg/dL)	1.0 (0.6; 3.7)	0.3–1.2
Creatinin (mg/dL)	1.1 (0.6; 5.0)	0.9–1.3
Albumin serum (g/dL)	2.3 (2.1; 2.8)	3.4–4.8
CRP (mg/dL)	1.3 (0.5; 13.7)	<0.5
Ascites total protein (mg/dL)	2086 (461; 3062)	-
Indication for TIPS variceal bleeding [high urgent procedure] refractory ascites [elective procedure]	3 (23%) 10 (77%)	-

^§^ Model of End Stage Liver Disease; ^‡^ activated partial thromboplastin time; ^†^ International Normalized Ratio.

**Table 2:** Rotational Thromboelastometry (ROTEM^®^) measurements shown as median (min; max).

EXTEM Value	MCV	HV/ICV	PV	TIPS	Normal Range
**CT, s**	72 (57; 104)	69 (57; 100)	70 (59; 93)	74 (59; 110)	35–80
**CFT, s**	98 (78; 486)	136 (78; 321)	106 (75; 316)	112 (79; 368)	35–160
**MCF, mm**	57 (33; 66)	53 (37; 64)	57 (39; 66)	56 (37; 65)	53–72
**ML, %**	10 (4; 17)	12 (3; 19)	9 (3; 15)	9 (4; 16)	<15

MCV: median cubital vein; HV/ICV: confluence of three hepatic veins “HV” to the inferior cava vein “ICV”; PV: portal vein; TIPS: transjugular intrahepatic portosystemic shunt; CT: clotting time; CFT: clot formation time; MCF: maximum clot firmness; ML: maximum lysis; s: seconds; mm: millimeters.

## Data Availability

The data that support the findings of this study are available from the corresponding author, upon reasonable request.
